# A Meta-Analysis Examining the Impact of Consuming Nitrogen-Free Analogs of Essential Amino Acids on the Progression of Chronic Renal Disease

**DOI:** 10.3390/medicina61030423

**Published:** 2025-02-28

**Authors:** Mohamed S. Imam, Lama Saud Turki Alrasheedi, Saleh Ali Hassan Alyami, Mahdi Mohammed Ahmed Aljamaan, Khaled Sami Khaled Alnaim, Hussam Mohsen Ayesh Alenzi, Nouf Nawaf Alnufeai, Daad Adnan Saad Almalki, Abdullah S. Alanazi, Saud Saad Frais Alotaibi, Naif Fahad Mashaan Alshaibani, Mohamed E. A. Abdelrahim, Basma M. E. Mohamed

**Affiliations:** 1 Department of Clinical Pharmacy, National Cancer Institute, Cairo University, Fom El Khalig Square, Kasr Al-Aini Street, Cairo 11796, Egypt; imammohamed311@gmail.com; 2 College of Pharmacy, Northern Border University, Rafha 91911, Saudi Arabia; saudlama2@gmail.com; 3 College of Clinical Pharmacy, King Faisal University, Al-Ahsa 31982, Saudi Arabia; saleh.boy1088@gmail.com (S.A.H.A.); mahdi.aljamaan03@gmail.com (M.M.A.A.); khaled.sami.alnaim@gmail.com (K.S.K.A.); hosamalenzy1@gmail.com (H.M.A.A.); 4 College of Pharmacy, Taif University, Taif 21944, Saudi Arabia; noufeai2000@gmail.com (N.N.A.); daad.almalki@gmail.com (D.A.S.A.); 5 Medical College School of Health and Social Care, Swansea University, Swansea SA1 8EN, UK; abdullah.salem.k@gmail.com; 6 College of Pharmacy, Shaqra University, Shaqra 11961, Saudi Arabia; iiu31286@gmail.com (S.S.F.A.); naiffahadm1234@gmail.com (N.F.M.A.); 7 Clinical Pharmacy Department, Faculty of Pharmacy, Beni-Suef University, Beni-Suef 62574, Egypt; mohamed.abdelrahim@pharm.bsu.edu.eg

**Keywords:** very-low-protein diet, chronic kidney disease, nitrogen-free analogs, nutrition status, kidney functions, conventional low-protein diet

## Abstract

*Background and Objectives*: We conducted a meta-analysis to assess the impact of nitrogen-free substitutes for essential amino acids on the progression of chronic kidney disease (CKD). *Materials and Methods*: A comprehensive literature review conducted up to November 2024 identified 15 studies that involved 1596 participants with CKD at baseline; among them, 797 were on very-low-protein diets (LPDs) enriched with nitrogen-free analogs (NFA), while 799 followed a standard LPD. *Results*: A very-LPD utilizing NFA showed significantly improved estimated glomerular filtration rate (MD, 1.00; 95% CI, 0.35–1.64, *p* = 0.002), reduced serum creatinine (MD, −0.44; 95% CI, −0.75 to −0.13, *p* = 0.006), decreased blood urea nitrogen (MD, −35.34; 95% CI, −64.27 to −6.42, *p* = 0.02), and lower parathyroid hormone levels (MD, −1.25; 95% CI, −2.33 to 0.18, *p* = 0.02) when compared to a standard LPD in patients with CKD. Nevertheless, the very-LPD with NFA resulted in no significant differences in serum albumin (MD, 0.08; 95% CI, −0.03 to 0.19, *p* = 0.14), serum cholesterol (MD, −17.25; 95% CI, −42.79 to 8.29, *p* = 0.19), serum phosphorus (MD, −0.41; 95% CI, −0.97 to 0.15, *p* = 0.15), and serum calcium (MD, 0.16; 95% CI, −0.06 to 0.39, *p* = 0.16) compared to a typical LPD in subjects with CKD. *Conclusions*: A very-LPD supplemented with NFA showed a notably higher estimated glomerular filtration rate, decreased serum creatinine levels, lower blood urea nitrogen, and reduced parathyroid hormone levels; however, there were no significant differences observed in serum albumin, serum cholesterol, serum phosphorous, and serum calcium when compared to a standard LPD in individuals with CKD. Additional research is necessary to confirm these results.

## 1. Introduction

Chronic kidney disease (CKD) is a significant public health concern worldwide. The absence of an appropriate low-protein diet (LPD) in individuals with CKD may lead to end-stage renal disease. The objective of managing CKD is to stop or slow auxiliary harm to the kidneys. Nutritional adjustments are the fundamental methodology when it comes to managing kidney failure [1]. The primary dietary change needed in CKD involves managing protein-related yields that contribute to acidemia, hyperphosphatemia, and hyperazotemia. Additionally, sodium and phosphate are significant in renal adaptation, resulting in hyperparathyroidism and an increase in extracellular volume, respectively [2]. Typically, dietary management of CKD should ensure a balanced intake of protein, phosphorus, potassium, sodium, fluid, and energy, taking into account biochemical markers and changes in weight [3]. Ketoanalogues of amino acids refer to nitrogen-free variants of essential amino acids (EAAs). The term “Keto diet” denotes a dietary regimen involving Ketoanalogues of amino acids paired with LPDs (0.6 g/kg/day) or very-LPDs (0.3–0.4 g/kg/day), facilitating diminished nitrogen consumption while mitigating the adverse consequences linked to insufficient dietary protein and malnutrition [4]. Research indicates that these diets can significantly reduce renal death in well-nourished individuals with progressive CKD, provided they adhere to the diet and have low co-morbidity [5]. A meta-analysis involving individuals with stage 3–5 CKD who had not yet started conservation dialysis indicated that a limited-protein diet enhanced with nitrogen-free amino acid analogs can postpone the progression of CKD, reduce hyperparathyroidism, hyperphosphatemia, and assist in maintaining blood pressure levels typical of an LPD, all without causing malnutrition [6]. There are a limited number of studies that examine the effects of early interventions utilizing nitrogen-free analogs (NFAs) of amino acids on nutritional status and mineral and bone-related disorders. This research seeks to evaluate the influence of a diet composed of NFAs of essential amino acids (EAAs) on the advancement of chronic kidney disease (CKD).

## 2. Materials and Methods

### 2.1. Eligibility Criteria

The present meta-analysis complies with the epidemiology statement’s meta-analysis of papers (PRISMA) [7]. This was carried out by a set protocol.

### 2.2. Information Sources

**Figure 1:**
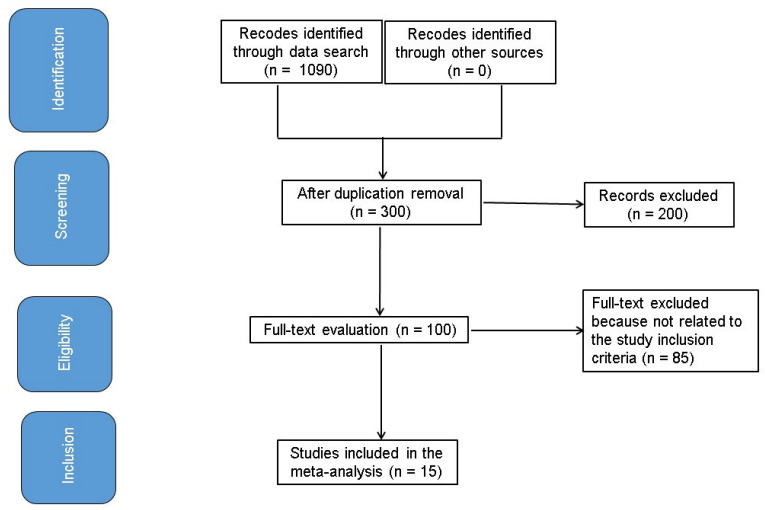
Schematic illustration of the study method.

The studies included in this analysis examined the statistical relationships regarding the impact of a diet consisting of NFAs of EAAs on the progression of CKD. Only human studies, regardless of language, were deemed eligible for selection. The inclusion criteria were not constrained by the type or size of study. Review articles, commentaries, and studies lacking a defined level of connotation were excluded. Figure 1 illustrates the comprehensive formula utilized in this study. The articles were incorporated into meta-analysis when the following criteria were satisfied:

1. The research was a randomized controlled trial, a prospective study, or a retrospective study.
2. The goal populace comprised persons with CKD.
3. The intervention consisted of a diet featuring NFAs of EAAs.
4. The study involved comparisons between very-LPDs that were supplemented with NFA and standard LPDs.

The exclusion criteria were as follows:

1. Studies that failed to assess the effect of the diet involving NFAs of EAAs on the deterioration of CKD.
2. Studies involving subjects receiving interventions other than a diet of NFAs of EAAs.
3. Studies that did not emphasize the impact of comparative results.

### 2.3. Search Strategy

A search plan protocol was established using the PICOS framework, which we defined as follows: P (population): individuals with CKD; I (intervention/exposure): consumption of NFA of EAAs; C (comparison): very-LPD with NFA versus traditional LPD; O (outcome): estimated glomerular filtration rate (EGFR), serum creatinine levels (SCLs), blood urea nitrogen (BUN), serum albumin concentration (SAC), serum cholesterol levels, serum phosphorus, serum calcium, parathyroid hormone (PH), and nutritional status; and S (study design): no restrictions [8]. Initially, we conducted a comprehensive search across PubMed, Embase, Google Scholar, Cochrane Library, and OVID up to November 2024, utilizing a combination of keywords and terms related to chronic kidney disease, very-low-protein diet, nitrogen-free analogs, kidney functions, nutritional status, and a conventional low-protein diet. All of the studies identified were organized into an EndNote file, duplicates were eliminated, and titles and abstracts were examined to filter out studies that did not demonstrate any link between the effects of NFAs of EAAs and the progression of CKD. The studies that remained were analyzed for relevant information.

### 2.4. Selection Process

The primary outcome focused on how a diet consisting of nitrogen-free substitutes for EAAs influences the progression of CKD. A summary was created by extracting the evaluation of the impact of this diet on the worsening of CKD.

### 2.5. Data Collection Process

Data were condensed based on the following criteria: study-connected and subject-connected characteristics. They were transformed into a consistent format, including the primary author’s last name, study duration, year of publication, country, population type, study design, and study region; along with the total number of participants, demographic information, and clinical and treatment details. Furthermore, the evaluation time corresponds with the measurement approach, employing both quantitative and qualitative assessment methods, sources of information, outcome evaluation, and statistical analyses such as mean difference (MD) or relative risk, along with a 95% CI for the connection [8].

### 2.6. Data Items

When a study met the criteria for inclusion based on the previously mentioned principles, two authors independently extracted the data. If there were discrepancies, the corresponding author made the final decision. In cases where a single study provided different data regarding the impact of a diet consisting of NFAs of EAAs on the progression of CKD, we extracted the data separately.

### 2.7. Study Risk of Bias Assessment

The potential for bias in these studies was measured by two authors, who separately assessed the methodological quality of the selected investigations. We employed the “risk of bias tool” from RoB 2: A Revised Cochrane Risk-of-Bias instrument for Randomized Trials to assess methodological quality. Each study was evaluated and categorized into one of three categories of bias risk based on the assessment criteria. The study was classified as having a low risk of bias if all quality criteria were met. If one or more quality criteria were partially met or uncertain, the study was assessed as having a moderate risk of bias. If one or more criteria were unmet or absent, the study was classified as having a high risk of bias. Discrepancies were rectified through an examination of the source article.

### 2.8. Effect Measures

The sensitivity analyses were confined to studies that illustrated the correlation between the NFA of EAAs in diets and the advancement of CKD. For the subgroup and sensitivity analysis, we accomplished a comparison between very-low-protein diets (LPDs) that incorporated NFAs and regular LPDs.

### 2.9. Synthesis Methods

We calculated the MD and the 95% CI using a contentious technique with either a random or fixed-effect model. We also computed the I^2^ index, which ranged from 0% to 100%. When the I² index fell around 0%, 25%, 50%, and 75%, it indicated no, low, moderate, and high heterogeneity, respectively. If the I² exceeded 50%, we applied the random-effect model; if it was below 50%, we utilized the fixed-effect model. To conduct subgroup analysis, we stratified the original calculations based on the previously defined result categories. A *p*-value of less than 0.05 for differences among subgroups indicated statistical significance.

### 2.10. Reporting Bias Assessment

We assessed study bias quantitatively using the Egger regression test (study bias is evident if *p* ≥ 0.05) and qualitatively through a visual inspection of funnel plots displaying the logarithm of mean differences against their standard errors.

### 2.11. Certainty Assessment

All *p*-values were two-tailed. All measurements and graphs were produced using Reviewer Manager version 5.3 (The Nordic Cochrane Centre, The Cochrane Collaboration, Copenhagen, Denmark).

## 3. Results

This meta-analysis, which encompasses 15 studies conducted between 1980 and 2023, consists of 1596 participants diagnosed with CKD at the outset. Of these participants, 797 followed very-LPDs that were enhanced with NFAs, while 799 adhered to conventional LPDs, as shown in Table 1 [5,9,10,11,12,13,14,15,16,17,18,19,20,21,22].

**Figure 2:**
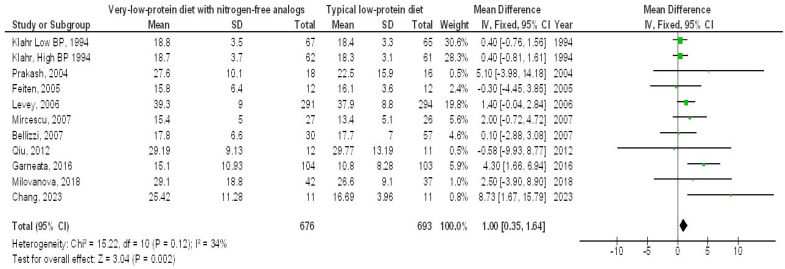
A forest plot illustrating the effects of very-low-protein diets with nitrogen-free analogs on the estimated glomerular filtration rate, in contrast to conventional low-protein diets in individuals with chronic kidney disease [5,10,13,14,15,16,17,18,19,22].

**Figure 3:**
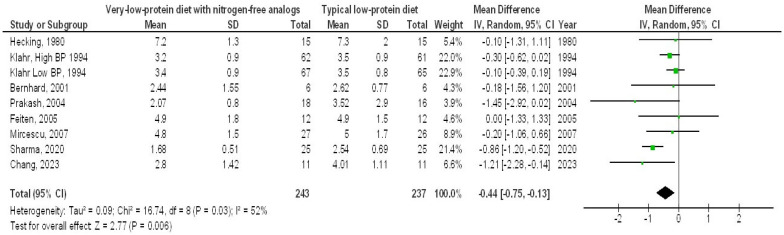
A forest plot illustrating the efficacy of very-low-protein diets with nitrogen-free analogs on blood creatinine levels, in comparison to standard low-protein diets in individuals with chronic renal disease [9,10,12,13,14,17,20,22].

**Figure 4:**
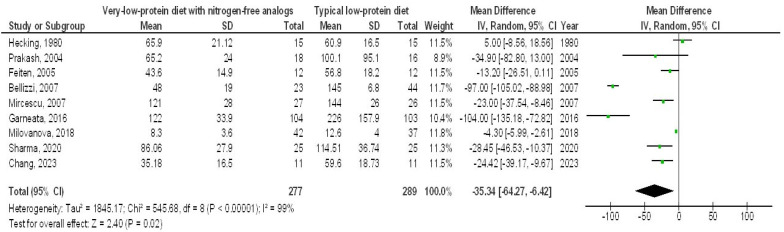
A forest plot illustrating the efficacy of very-low-protein meals with nitrogen-free analogs on blood urea nitrogen levels, in comparison to standard low-protein diets in individuals with chronic renal disease [5,9,13,14,16,17,19,20,22].

**Figure 5:**
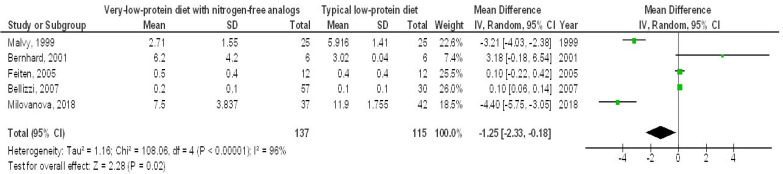
A forest plot illustrating the efficacy of very-low-protein meals with nitrogen-free analogs on parathyroid hormone levels, in comparison to standard low-protein diets in individuals with chronic renal disease [11,12,14,16,19].

A very-LPD featuring NFA demonstrated a significantly higher EGFR (MD, 1.00; 95% CI, 0.35–1.64, *p* = 0.002) with low heterogeneity (I^2^ = 34%), as well as a lower SCL (MD, −0.44; 95% CI, −0.75 to −0.13, *p* = 0.006) with moderate heterogeneity (I2 = 52%), a reduced BUN (MD, −35.34; 95% CI, −64.27 to −6.42, *p* = 0.02) showing high heterogeneity (I2 = 99%), and lower PH levels (MD, −1.25; 95% CI, −2.33 to 0.18, *p* = 0.02), also with high heterogeneity (I2 = 96%), in contrast to the typical LPD among individuals with CKD, as illustrated in Figure 2, Figure 3, Figure 4 and Figure 5.

**Figure 6:**
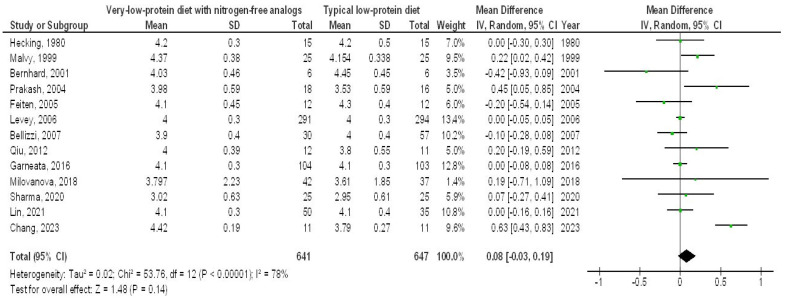
A forest plot illustrating the effects of very-low-protein meals with nitrogen-free analogs on serum albumin levels, in comparison to standard low-protein diets in individuals with chronic renal disease [5,9,11,12,13,14,15,16,18,19,20,21,22].

**Figure 7:**
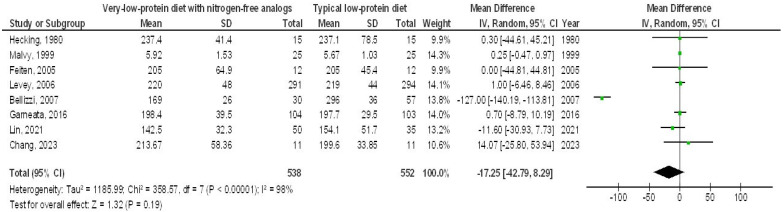
A forest plot illustrating the efficacy of very-low-protein diets with nitrogen-free analogs on blood cholesterol, compared to standard low-protein diets in individuals with chronic renal disease [5,9,11,14,15,16,21,22].

**Figure 8:**
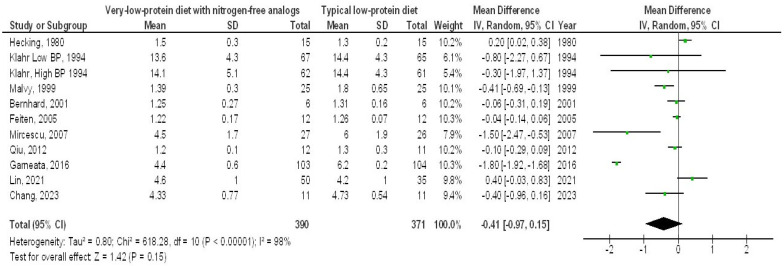
A forest plot illustrating the effects of very-low-protein diets with nitrogen-free analogs on serum phosphorus levels, in comparison to standard low-protein diets in individuals with chronic renal disease [5,9,10,11,12,14,17,18,21,22].

**Figure 9:**
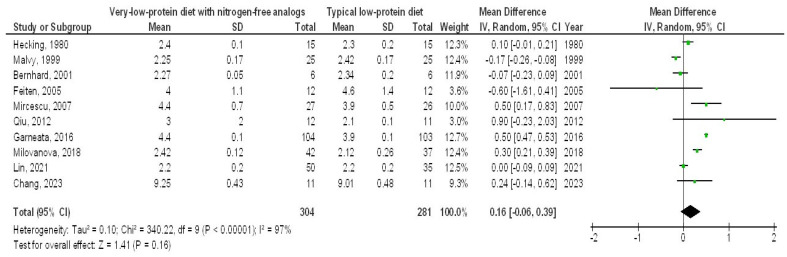
A forest plot illustrating the efficacy of very-low-protein diets with nitrogen-free analogs on serum calcium levels, in comparison to standard low-protein diets in individuals with chronic renal disease [5,9,11,12,14,17,18,19,21,22].

In contrast, the very-LPD with NFAs exhibited no significant differences in SAC (MD, 0.08; 95% CI, −0.03 to 0.19, *p* = 0.14) with high heterogeneity (I2 = 78%), serum cholesterol (MD, −17.25; 95% CI, −42.79 to 8.29, *p* = 0.19) with high heterogeneity (I2 = 98%), serum phosphorus (MD, −0.41; 95% CI, −0.97 to 0.15, *p* = 0.15) with high heterogeneity (I2 = 98%), and serum calcium (MD, 0.16; 95% CI, −0.06 to 0.39, *p* = 0.16) with high heterogeneity (I2 = 97%) when compared to the typical LPD in CKD patients, as depicted in Figure 6, Figure 7, Figure 8 and Figure 9.

An analysis of chosen studies that accounted for ethnicity and age adjustments was not performed due to the absence of research reporting or adjusting for these variables. Both the visual analysis of the funnel plot and the quantitative evaluation via the Egger regression test revealed no evidence of publication bias (*p* = 0.91). Nonetheless, the majority of the studies included had inadequate methodological quality due to their limited sample sizes. All research mitigated selective reporting bias, and there were no articles with incomplete result data or selective reporting.

## 4. Discussion

This meta-analysis, which encompasses 15 studies, consisted of 1596 participants diagnosed with CKD at the outset. Of these participants, 797 followed very-LPDs that were enhanced with NFA, while 799 adhered to conventional LPDs [5,9,10,11,12,13,14,15,16,17,18,19,20,21,22]. A very-LPD incorporating NFA showed a significantly higher EGFR, along with lower serum creatinine, reduced BUN, and decreased PH levels compared to a typical LPD in individuals with CKD. However, there were no significant differences in SAC, serum cholesterol, serum phosphorus, or serum calcium between the very-LPD with NFA and the typical LPD among subjects with CKD. Nonetheless, one must cautiously interpret these results due to the small sample sizes in many of the studies included in the meta-analysis, with 12 out of 15 studies having a sample size of 100 subjects or fewer, along with a limited number of studies assessing certain parameters like PH, highlighting the need for further research to validate these findings and potentially enhance confidence in the calculation of effects. This meta-analysis consolidated the evidence regarding the efficacy of NFA of EAAs in dietary management for individuals with CKD. The burden of CKD and its related co-morbidities, such as cardiovascular diseases, mineral and bone disorders, and anemia, has created a significant health challenge globally [23,24,25,26,27]. The progression of CKD ultimately causes end-stage renal disease, for which there are currently no medical treatments available [28]. The prevention of CKD progression is a crucial aspect of enhancing the longevity of individuals with CKD. Current approaches to managing CKD are predominantly conservative, focusing on postponing the need for dialysis and addressing signs and symptoms caused by related co-morbidities. Ketosteril^®^ is a nitrogen-free alternative to EAA formulations that may help slow the progress of CKD. However, there is a deficiency of solid indication demonstrating the effectiveness of these nutritional supplements. Nutritional deficiencies pose a significant issue for individuals with CKD. Restricting protein intake can protect the kidneys and slow the advancement of CKD by decreasing albumin levels and reducing renal fibrosis [29,30,31]. However, these diets could lead to nutritional deficiencies [29] or contribute to nutrition-related co-morbidities such as metabolic acidosis [32,33], hormone disorders [34], inflammation [35], and the depletion of protein and energy resources. Research focused on dietary alterations in people with renal disease indicated that in long-term observations, an extremely LPD did not postpone the onset of kidney failure, and it appeared to elevate the risk of death [36]. However, there was an absence of evaluations related to dietary protein during the follow-up [36].

Utilizing cholesterol and albumin as indicators of nutritional status may be debatable and subject to inconsistency [37]. Using muscle mass, dietary consumption, and nutritional scoring systems to evaluate nutritional status and body mass instead of relying solely on albumin or cholesterol is indeed a more effective, meaningful, and appropriate approach [38]. However, there are still discussions supporting the straightforward use of albumin as a nutritional indicator for assessing kidney disorders [39]. A significant number of papers in this meta-analysis lacked comprehensive details on characteristics like fat and carbohydrate percentages, complicating the evaluation of these components. Moreover, assessing albumin and cholesterol levels in people with CKD is more practical for evaluating their nutritional health, especially in areas with constrained medical resources or developing nations. The Kidney Disease Improving Global Outcomes group defines CKD–mineral and bone disorder as a condition that affects mineral and bone metabolism as a result of CKD. These conditions are characterized by abnormalities in phosphorus, calcium, PH, or vitamin D metabolism; disruptions in bone mineralization, turnover, linear growth, strength, volume; or calcification of blood vessels and other soft tissues [25,40]. Previous research indicated that elevated serum phosphorus levels, increased calcium–phosphorus production, or high PH levels are connected with worse clinical outcomes and an increased risk of death in individuals with CKD [40,41,42,43]. Researching the treatment of mineral and bone disorders connected to CKD is essential. However, the effectiveness of NFAs of EAAs in reversing CKD-related mineral and bone disorders is still insufficient [5,11,12,18]. Jiang et al. demonstrated that nitrogen-free versions of EAAs meaningfully lowered serum phosphorus and PH levels without influencing calcium levels [6]. The primary use of NFAs of EAAs has shown effectiveness in reversing CKD–mineral and bone disorder; however, a statistically significant difference is only apparent when the severity of the condition is high. This meta-analysis examined the relationship between a diet containing NFAs of EAAs and the progression of CKD. Nevertheless, further research is desired to confirm these possible connotations. Moreover, additional studies should aim to demonstrate clinically significant differences in outcomes. This need for further research was also highlighted in previous meta-analyses, which indicated comparable effects of very-LPDs supplemented with NFA and standard LPDs among patients with CKD [6,44,45]. The negligible findings regarding the diet of nitrogen-free substitutes for EAAs on SAC, serum cholesterol, serum phosphorus, serum calcium, and PH, in comparison to a typical LPD, necessitate additional research and explanation, as no definitive reasoning was discovered to clarify these results. Well-designed studies are also necessary to examine these factors alongside variations in age and ethnicity, as our meta-analysis was unable to determine whether these were connected to the outcomes. We recommend that high-quality, well-structured randomized control trials be conducted to investigate the impact of NFA of EAAs on individuals with CKD. Healthcare professionals should ensure that completed research is published to record and validate findings concerning the dietary effects of NFA of EAAs on patients with CKD, as published evidence should inform clinical practice [46].

### Limitations

This research may exhibit selection bias due to the exclusion of numerous studies from the meta-analysis. Discarded studies failed to fulfill inclusion criteria for meta-analysis. Furthermore, we could not ascertain if the results were affected by ethnicity and age. The purpose of the study was to measure the connection between the impact of a diet consisting of NFAs of EAAs and care outcomes for individuals with CKD, drawing from data from previous studies, which may introduce bias due to insufficient information. The meta-analysis included only 15 studies; 12 of these were small, with sample sizes of 100 or less. Factors such as the ethnicity, nutritional status, and age of participants might also contribute to potential biases. The presence of some unpublished articles and omitted information could create bias in the combined results. Participants were subjected to various management plans, dosages, and healthcare institutions. The duration of the dietary management involving NFA of EAAs varied across the studies included. It should be noted that it is difficult to adjust for confounding factors such as baseline nutritional status as a limitation.

## 5. Conclusions

A very-LPD supplemented with NFA resulted in a significantly higher EGFR, lower SCLs, reduced BUN, and decreased PH levels compared to a typical LPD in individuals with CKD. However, there was no significant difference in SAC, serum cholesterol, serum phosphorus, or serum calcium levels between the very-LPD with NFA and the typical LPD in individuals with CKD.

## Figures and Tables

**Table 1:** Characteristics of the studies selected for the meta-analysis.

Study	Country	Subjects	Age (Years)	Study Design	Treatment Period	Main Results
Hecking, 1980 [9]	Germany	30 (15/15)	very-LPD: 43.7 ± 12.6 LPD: 43.7 ± 12.6	RCT; 1.05 g/10 kg/day NFA vs. LPD (0.60 g/kg/d)	6 weeks	SCL BUN SAC SC SP C
Klahr, 1994 [10]	USA	255 (129/126)	NA	RCT; 0.28 g/kg/day NFA vs. LPD (0.60 g/kg/d)	18–45 months	EGFR SCL SP
Malvy, 1999 [11]	France	50 (25/25)	very-LPD: 53.6 ± 11.0 LPD: 56.0 ± 14.0	RCT; 0.17 g/kg/day NFA vs. LPD (0.58 g/kg/d)	3 months	PH SAC SC SP C
Bernhard, 2001 [12]	France	12 (6/6)	very-LPD: 49.5 ± 7.0 LPD: 39.0 ± 5.8	RCT; 1 pill/5 kg/day NFA vs. LPD RCT; (0.60–80 g/kg/d)	3 months	SCL PH SAC SP C
Prakash, 2004 [13]	India	34 (18/16)	very-LPD: 52.8 ± 14.1 LPD: 55.9 ± 17.6	RCT; 1 pill/5 kg/day NFA vs. LPD (0.60 g/kg/d)	9 months	EGFR SCL BUN SAC
Feiten, 2005 [14]	Brazil	24 (12/12)	very-LPD: 49.7 ± 11.3 LPD: 43.9 ± 16.3	RCT; 1 pill/5 kg/day NFA vs. LPD (0.60 g/kg/d)	4 months	EGFR SCL BUN PH SAC SC SP C
Levey, 2006 [15]	USA	585 (291/294)	NA	RCT; 1.05 g/10 kg/day NFA vs. LPD (0.65 g/kg/d)	36 months	EGFR SAC SC
Bellizzi, 2007 [16]	Italy	87 (30/57)	very-LPD: 58.0 ± 16.1 LPD: 56.3 ± 15.6	RCT; 1 pill/5 kg/day NFA vs. LPD (0.60–80 g/kg/d)	3–6 months	EGFR BUN PH SAC SC
Mircescu, 2007 [17]	Romania	53 (27/26)	very-LPD: 55.0 ± 12.7 LPD: 53.6 ± 11.0	RCT; 1 pill/5 kg/day NFA vs. LPD (0.60–80 g/kg/d)	15 months	EGFR SCL BUN SP C
Qiu, 2012 [18]	China	23 (12/11)	very-LPD: 63.0 ± 8.9 LPD: 61.60 ±9.67	RCT; 1 pill/5 kg/day NFA vs. LPD (0.60–80 g/kg/d)	52 months	EGFR SAC SP C
Garneata, 2016 [5]	Romania	207 (104/103)	NA	RCT; 1 pill/5 kg/day NFA vs. LPD (0.60 g/kg/d)	12 months	EGFR BUN SAC SC SP C
Milovanova, 2018 [19]	Russia	79 (42/37)	NA	RCT; 0.1 g/kg/day NFA vs. LPD (0.60 g/kg/d)	14 months	EGFR BUN PH SAC C
Sharma, 2020 [20]	Nepal	50 (25/25)	very-LPD: 42.3 ± 13.6 LPD: 41.9 ± 11.6	RCT; 1 pill/5 kg/day NFA vs. LPD (0.60 g/kg/d)	24 months	SCL BUN SAC
Lin, 2021 [21]	Taiwan	85 (50/35)	NA	RCT; 1 pill/5 kg/day NFA vs. LPD (0.58 g/kg/d)	15 months	SAC SC SP C
Chang, 2023 [22]	Taiwan	22 (11/11)	very-LPD: 45.6 ± 11.0 LPD: 44.8 ± 14.5	RCT; 1 pill/5 kg/day NFA vs. LPD (0.65 g/kg/d)	36 months	EGFR SCL BUN SAC SC SP C
	Total	1596 (797/799)				

Blood urea nitrogen (BUN); glomerular filtration rate (EGFR); low-protein diets (LPDs); nitrogen-free analogs (NFAs); parathyroid hormone (PH); serum albumin concentration (SAC); serum calcium (C); serum cholesterol (SC); serum creatinine level (SCL); serum phosphorus (SP).
